# Environmental factors impacting the motivation to innovate: a systematic review

**DOI:** 10.1186/s13731-021-00153-9

**Published:** 2021-06-30

**Authors:** Eleftherios Soleas

**Affiliations:** Faculty of Education, Queen’s University, Kingston, Canada

**Keywords:** Innovation, Environment, Motivation, Expectancy-value, Innovation education, Systematic reviews, Interdisciplinary approaches

## Abstract

The environments where innovation occurs are often as varied as the areas of endeavors that aspiring innovators could pursue. This systematic review followed the guidelines of the Campbell Collaboration and PRISMA to consolidate the findings of 74 studies into the Expectancy-Value-Cost motivation theoretical framework as a means of usefully isolating for decision-makers the environmental factors that impact the motivation to innovate. The results of this review reveal that additional study of interdisciplinary samples is needed to gather deep narrative and case-driven data that considers the experiences of innovators in addition to organizations. Leaders, including decision-makers, teachers, and supervisors, can set a precedent for their learners and workers to use their past experiences and to feel safe to take intelligent risks and make reasonable mistakes in pursuit of innovating. Ensuring that project teams have a mix of experiences and backgrounds can make for more productive collaborations. Proactively addressing costs can increase workplaces’ psychological safety and stability, which enables workers and learners to better focus on the endeavors at hand. The articles’ evaluation illustrates that conversation about innovation promotion is dominated by business, which reduces the opportunity to learn from other innovation-driven disciplines or take truly interdisciplinary approaches.

## Introduction

Innovation has diverse conceptualizations and foci in different contexts, such as generating wealth through ideas driven by entrepreneurship in business settings and applying creativity in psychology (Baregheh et al., 2009; Carayannis et al., 2018). The amorphous meaning of the word causes problems with a unified definition of innovation for society and knowing who exactly innovators are and what can be done to support them (Arafeh, 2015; Johannessen et al., 2001; Nager et al., 2016; Soleas, 2018; U.S. Department of Education, 2014). Therefore, it is important for society’s progression that students, workers, and leaders (henceforth all referred to as learners) live and work in environments supportive of their innovating. These environments are often as varied as the areas of endeavors that aspiring innovators could pursue. This article aims to consolidate the available knowledge in the literature to comprehensively identify the ways that environment can contribute to the motivation to innovate. It does so through a systematic review methodology and uses the Expectancy-Value-Cost Theory framework (Barron & Hulleman, 2015) to organize the findings to comprehensively answer the research questions.

Expectancy-Value-Cost Theory (EVC) is a motivation framework that conceptualizes the motivation to complete a task as an interaction of the factors that make it more likely arrayed against those that actively detract from the motivation to complete the task in question. Expectancies (e.g., built expectations of success, perceived self-efficacy, and acquired confidence) and the subjective task values (interest, fulfillment, utility) are the promotive factors, while “perceived costs” are the hindering factors that make it less likely that a task is completed such as stress, financial considerations, external pressures, and the implications of failing at the task (Flake et al., 2015).

This dynamic presents an opportunity to ascertain the characteristics of working and learning environments that influence the motivation to innovate. For instance, what learning or work environments increase the confidence of learners for innovating? What structural or environmental factors can reduce the costs of innovating to the point where more learners are willing to give it a try? This two-pronged approach of evaluating the literature offerings on both innovation-promotive and hindering motivation factors would provide an interdisciplinary view of the environmental factors that could make innovation more likely and approach hindering factors that need to be addressed.

Systematic reviews are structured literature reviews where researchers retrieve all available evidence on a given topic; they synthesize, categorize, and appraise all the actual knowledge pertaining to a topic of inquiry (Heyvaert et al., 2013; Liberati et al., 2009; The Campbell Collaboration, 2017). In this case, the review sought all articles through an EBSCOhost all database search on environmental for promoting individuals’ capacity to innovate. A systematic review is an ideal methodology for consolidating knowledge from a varied range of disciplines (Liberati et al., 2009; The Campbell Collaboration, 2017), albeit an uncommon one in a domain with as much interdisciplinary interest as motivating innovation, which has made disciplinary siloing a far more academically comfortable outcome (Soleas, 2020).

## Methodology

In searching, collecting, and reviewing literature for this study, the Campbell Collaboration’s protocols (The Campbell Collaboration, 2017), as well as the PRISMA Statement for systematic reviews (Liberati et al., 2009), were closely followed to ensure that the required information was provided for rigor and replicability such as databases searched, search terms, and the dates when searches were conducted. In this case, the registered systematic review (osf.io/up83s) consolidated and analyzed empirical studies through an EBSCOhost all database search of peer-reviewed scholarly contributions (books, articles, chapters, peer-reviewed conference proceedings) about the environments, which includes context, cultural, and situational factors, guided by the following research questions:

1. What does the existing literature tell us about environments that motivate and sustain human innovative behavior?
  - What is found in the literature about environments that build expectations of success and self-efficacy of individual innovative behavior?
  - What is found in the literature about environments that build subjective task values for individual innovative behavior?
  - What is found in the literature about environments that mitigate the perceived costs of individual innovative behavior?

Specific consideration was given to the design of environments that build human expectancies and help mitigate the perceived and unperceived costs or risks of innovative behavior. This systematic review followed an all database (*n*=375) search through EBSCOhost initially performed on January 2, 2018, and expanded upon on April 6, 2019, in addition to Google and Bing search engine use. The latter review captured all the previous review contributions but with the benefit of additional articles published in the elapsed time.

A challenge with the current literature on innovation and a key reason why a systematic review is necessary is the nebulous and often conjectural ideas defining and explaining innovators’ motivation. Innovation is deeply entangled with concepts such as invention, entrepreneurship, and novelty (Carayannis et al., 2018; Weisenfeld & Hauerwaas, 2018). To manage this issue, only empirical studies specific to innovation, with human participants that examined individuals’ motivations as a unit of analysis, were included. Additionally, only English-language articles and articles with verified English-language translations were considered. There were no restrictions on the years of included studies, with the earliest studies dating back to 1967 and the most recent published in early 2019.

**Fig. 1:**
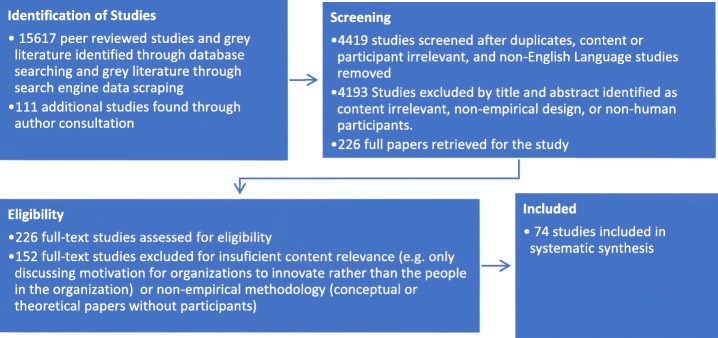
Flow of information in this systematic review study

Similarly, when contacting authors of the included papers for additional sources to consider, there were no date-of-publication restrictions. Seventy-eight studies from outside the database search were obtained this way, while an additional 64 studies were found to be already included in the search and removed as redundant. Database searching was concluded on May 6, 2019, and the last of the article-yielding author replies was retrieved by May 8, 2019 (Fig. 1).

### Search strategy

In Abstract AND Paper: Innovat* AND (Motiv* OR Promot* OR Support*) AND (environ* OR climat* or context*). Related word substitutions are allowed.

As secondary search procedures, Google and Bing were also searched using an analogous process with Boolean search code operators. The authors’ email addresses were extracted, and these authors were contacted, resulting in 111 previously undiscovered prospective studies after eliminating redundant duplicates.

**Fig. 2:**
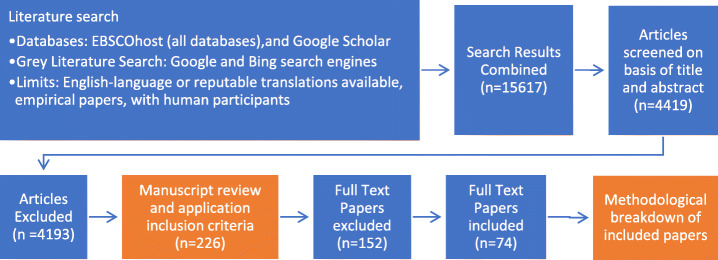
Flow diagram of the study selection

Research assistants were employed to facilitate the screening process by abstracts and titles. Study reviewers operated in dyads. Each member did independent reviews of each abstract and title by deciding whether they would be relevant and within the inclusion criteria. Each abstract and title was therefore reviewed twice to adjudicate inclusion or exclusion (Fig. 2). Disagreements were resolved through the study being included in the full paper review to avoid removing a potentially eligible study. Cohen’s Kappas were calculated using the tabulated data in the aggregate review (see Table 1). The Kappa value (0.895) indicates very good agreement among the reviewers. Two hundred twenty-six studies were full-text reviewed, of which 78 final studies were assessed to be fully eligible for inclusion in findings extraction.

**Table 1:** Screening phase: review of titles and abstracts—relative agreement as indicated by Cohen’s Kappa

Aggregate			Kappa = 0.895	99.07% agreement
	Reject	Keep	SE of Kappa = 0.016	91.13% of the agreement could be explained by chance
Reject	4193	18	95% CI = 0.864 to 0.927	
Keep	23	185	Rated as very good	

Findings were extracted from the final papers using qualitative data analysis software, Atlas.ti v8.0. In this way, salient hypotheses, methodologies, findings, conclusions, participant data, and other articles for consideration were isolated from the full-text PDFs. From the methodology, the paradigm (e.g., qualitative, quantitative, or mixed-methodology) and tradition (e.g., case study, experimental design, or pre-post-test) were isolated and extracted.

A few authors appeared on two manuscripts; however, these were very few and far between. There were no discernible patterns in the home institution of authors beyond a distinct prevalence of western institutions such as those in North America and Europe and far fewer from Africa, South America, and Asia, though we note that in recent years this trend is no longer quite as true. However, in terms of journal distribution of included articles, Frontiers in Psychology (5), Product Information Management (4), Occupational and Organization Psychology (2), Creativity Research Journal, Creativity and Innovation Management (2), Research Policy (2), and Industrial Marketing Management (2) were the only journals to have more than one article included in this synthesis.

Business workers and employees were the most common study participants (41.9%), followed by service consumers (17.6%), business leaders (13.5%), students (10.8%), entrepreneurs (8.11%), teachers (4.05%), higher education faculty (1.35%), libraries (1.35%), and astronauts (1.35%) (see Table 2 for a complete listing of studies by participant type). Many studies examined managers’ or leaders’ activities as crucial for promoting the innovative behavior of other workers. For simplicity’s sake, the term leader will be used for individuals who influence subordinate, worker, or learner groups in their care. In the case of schools, leaders would be those leading classes, namely, teachers and administrators.

**Table 2:** Study participant groupings of included studies

**Business employees**	**Business leaders**	**Consumers**
1. Aalbers et al. (2013)	1. Cordero et al. (2005)	1. Bolderdijk et al. (2018)
2. Aarikka-Stenroos et al. (2017)	2. Hosseini and Narayanan (2014)	2. Brandstätter (2011)
3. Amabile (1997)	3. Hsu (2009)	3. Costa et al. (2015)
4. Apergis and Pekka-Economou (2010)	4. Jermias (2007)	4. Duverger (2012)
5. Armstrong et al. (2018)	5. Liu and Chan (2017)	5. Füller et al. (2012)
6. Bergendahl et al. (2015)	6. Lukoschek et al. (2018)	6. Hasan et al. (2019)
7. Bessonova and Gonchar (2017)	7. Manimala et al. (2006)	7. Hopkins (2016)
8. Chen et al. (2019)	8. Naidoo and Sutherland (2016)	8. Kraft and Bausch (2018)
9. Chi et al. (2018)	9. Weisenfeld and Hauerwaas (2018)	9. Mc Fadden and Gorman (2016)
10. Curran and Walsworth (2014)	10. Wu et al. (2013)	10. Ng and Feldman (2013)
11. de Jong and Flowers (2018)	**Teachers**	11. Ozorhon and Oral (2017)
12. Delmas and Pekovic (2018)	1. Gorozidis and Papaioannou (2016)	12. Park et al. (2004)
13. Demircioglu and Audretsch (2017)	2. Lam et al. (2010)	13. Thapa et al. (2015)
14. Ederer and Manso (2013)	3. Messmann and Mulder (2014)	**Astronauts**
15. Fernandez and Pitts (2011)	**Entrepreneurs**	1. Brcic (2010)
16. Ford (1999)	1. Fischer et al. (2019)	**Students**
17. Galia (2008)	2. Griffin et al. (2009)	1. Bastian et al. (2018)
18. Gopal and College (2011)	3. Jiang and Thagard (2014)	2. Chis et al. (2018)
19. Hartmann (2006)	4. Shane et al. (2003)	3. Dietrich et al. (2016)
20. Lerner and Wulf (2018)	5. Susha et al. (2015)	4. Joy (2004)
21. Lettl (2007)	6. Todt et al. (2018)	5. Kung and Chao (2019)
22. Li and Yu (2018)	**Academics**	6. Mack and Landau (2015)
23. Maria Stock et al. (2017)	1. Kandiko (2013)	7. Pihie (2007)
24. Minarcine and Shaw (2016)	**Librarians**	8. Reznickova and Zepeda (2016)
25. Monge et al. (1992)	1. Koloniari et al. (2018)	
26. Montani et al. (2014)		
27. Öberg and Shih (2014)		
28. Pihlajamaa (2017)		
29. van Acker et al. (2018)		
30. Vansteenkiste et al. (2005)		
31. Wang et al. (2018)		

When comparing the results of analyzing articles by discipline, the disciplinary breakdown of articles mirrored the findings of Soleas (2018), including the disproportionate representation (55.3%) of innovation conceptualized in from business disciplines such as economics, management, and entrepreneurship compared to public sector studies (17.6%), construction and engineering (9.5%), and small minorities from primary and secondary education (6.8%), psychology (5.4%), higher education (4.1%), and design (1.3%) (see Table 3 for a complete study listing by discipline).

**Table 3:** Disciplinary groupings of included studies

**Business/management**		**Public sector**
1. Aarikka-Stenroos et al. (2017)	22. Hsu (2009)	1. Aalbers et al. (2013)
2. Amabile (1997)	23. Jermias (2007)	2. Demircioglu and Audretsch (2017)
3. Apergis and Pekka-Economou (2010)	24. Kung and Chao (2019)	3. Dietrich et al. (2016)
4. Armstrong et al. (2018)	25. Lerner and Wulf (2018)	4. Duverger (2012)
5. Bergendahl et al. (2015)	26. Li and Yu (2018)	5. Fernandez and Pitts (2011)
6. Bessonova and Gonchar (2017)	27. Lukoschek et al. (2018)	6. Hasan et al. (2019)
7. Brandstätter (2011)	28. Mack and Landau (2015)	7. Jiang and Thagard (2014)
8. Bolderdijk et al. (2018)	29. Maria Stock et al. (2017)	8. Kraft and Bausch (2018)
9. Chen et al. (2019)	30. Minarcine and Shaw (2016)	9. Manimala et al. (2006)
10. Chi et al. (2018)	31. Monge et al. (1992)	10. Mc Fadden and Gorman (2016)
11. Curran and Walsworth (2014)	32. Montani et al. (2014)	11. Thapa et al. (2015)
12. de Jong and Flowers (2018)	33. Naidoo and Sutherland (2016)	12. van Acker et al. (2018)
13. Delmas and Pekovic (2018)	34. Ng and Feldman (2013)	13. Vansteenkiste et al. (2005)
14. Ederer and Manso (2013)	35. Öberg and Shih (2014)	**Education**
15. Fischer et al. (2019)	36. Shane et al. (2003)	1. Chis et al. (2018)
16. Ford (1999)	37. Susha et al. (2015)	2. Gorozidis and Papaioannou (2016)
17. Füller et al. (2012)	38. Todt et al. (2018)	3. Lam et al. (2010)
18. Galia (2008)	39. Wang et al. (2018)	4. Messmann and Mulder (2014)
19. Gopal and College (2011)	40. Weisenfeld and Hauerwaas (2018)	5. Pihie (2007)
20. Griffin et al. (2009)	41. Wu et al. (2013)	**Higher education**
21. Hopkins (2016)		1. Kandiko (2013)
**Construction/engineering**	**Design**	2. Koloniari et al. (2018)
1. Cordero et al. (2005)	1. Lettl (2007)	3. Reznickova and Zepeda (2016)
2. Hartmann (2006)	**Psychology**	
3. Hosseini and Narayanan (2014)	1. Bastian et al. (2018)	
4. Liu and Chan (2017)	2. Brcic (2010)	
5. Ozorhon and Oral (2017)	3. Costa et al. (2015)	
6. Park et al. (2004)	4. Joy (2004)	
7. Pihlajamaa (2017)		

The vast majority of studies in the sample were quantitative (75.7%), with qualitative as a sizable minority (18.9%), and mixed method studies as the rarest (5.4%) (see Table 4 for a complete study listing by methodology type).

**Table 4:** Methodology type groupings of included studies

**Quantitative**		**Qualitative**
1. Aalbers et al. (2013)	29. Hosseini and Narayanan (2014)	1. Aarikka-Stenroos et al. (2017)
2. Amabile (1997)	30. Hsu (2009)	2. Armstrong et al. (2018)
3. Apergis and Pekka-Economou (2010)	31. Jermias (2007)	3. Brcic (2010)
4. Bastian et al. (2018)	32. Joy (2004)	4. Gopal and College (2011)
5. Bergendahl et al. (2015)	33. Koloniari et al. (2018)	5. Griffin et al. (2009)
6. Bessonova and Gonchar (2017)	34. Kraft and Bausch (2018)	6. Hartmann (2006)
7. Bolderdijk et al. (2018)	35. Kung and Chao (2019)	7. Jiang and Thagard (2014)
8. Brandstätter (2011)	36. Lam et al. (2010)	8. Kandiko (2013)
9. Chen et al. (2019)	37. Lerner and Wulf (2018)	9. Lettl (2007)
10. Chi et al. (2018)	38. Li and Yu (2018)	10. Manimala et al. (2006)
11. Chis et al. (2018)	39. Liu and Chan (2017)	11. Minarcine and Shaw (2016)
12. Cordero et al. (2005)	40. Lukoschek et al. (2018)	12. Naidoo and Sutherland (2016)
13. Costa et al. (2015)	41. Mack and Landau (2015)	13. Pihlajamaa (2017)
14. Curran and Walsworth (2014)	42. Maria Stock et al. (2017)	14. Reznickova and Zepeda (2016)
15.de Jong and Flowers (2018)	43. Messmann and Mulder (2014)	**Mixed**
16. Delmas and Pekovic (2018)	44. Monge et al. (1992)	1. Mc Fadden and Gorman (2016)
17. Demircioglu and Audretsch (2017)	45. Montani et al. (2014)	2. Ng and Feldman (2013)
18. Dietrich et al. (2016)	46. Ozorhon and Oral (2017)	3. Öberg and Shih (2014)
19. Duverger (2012)	47. Park et al. (2004)	4. Susha et al. (2015)
20. Ederer and Manso (2013)	48. Pihie (2007)	
21. Fernandez and Pitts (2011)	49. Shane et al. (2003)	
22. Fischer et al. (2019)	50. Thapa et al. (2015)	
23. Ford (1999)	51. Todt et al. (2018)	
24. Füller et al. (2012)	52. van Acker et al. (2018)	
25. Galia (2008)	53. Vansteenkiste et al. (2005)	
26. Gorozidis and Papaioannou (2016)	54. Wang et al. (2018)	
27. Hasan et al. (2019)	55. Weisenfeld and Hauerwaas (2018)	
28. Hopkins (2016)	56. Wu et al. (2013)	

In terms of research design, surveys were by far the most common design (63.5%), followed by case studies (12.2%), meta-analyses (8.1%), interviews (8.1%), experimental and quasi-experimental designs (6.7%), and then sequential explanatory mixed methods (1.35%) (see Table 5 for a complete listing by design.)

**Table 5:** Research design groupings of included studies

**Survey**		**Case study**
1. Aalbers et al. (2013)	25. Jermias (2007)	1. Aarikka-Stenroos et al. (2017)
2. Amabile (1997)	26. Joy (2004)	2. Brcic (2010)
3. Apergis and Pekka-Economou (2010)	27. Koloniari et al. (2018)	3. Chis et al. (2018)
4. Bergendahl et al. (2015)	28. Lam et al. (2010)	4. Hartmann (2006)
5. Bessonova and Gonchar (2017)	29. Lerner and Wulf (2018)	5. Jiang and Thagard (2014)
6. Chen et al. (2019)	30. Li and Yu (2018)	6. Lettl (2007)
7. Chi et al. (2018)	31. Liu and Chan (2017)	7. Manimala et al. (2006)
8. Cordero et al. (2005)	32. Lukoschek et al. (2018)	8. Öberg and Shih (2014)
9. Curran and Walsworth (2014)	33. Mack and Landau (2015)	9. Pihlajamaa (2017)
10. de Jong and Flowers (2018)	34. Maria Stock et al. (2017)	**Experimental design**
11. Delmas and Pekovic (2018)	35. Messmann and Mulder (2014)	1. Bastian et al. (2018)
12. Demircioglu and Audretsch (2017)	36. Monge et al. (1992)	2. Bolderdijk et al. (2018)
13. Dietrich et al. (2016)	37. Montani et al. (2014)	3. Duverger (2012)
14. Fernandez and Pitts (2011)	38. Ozorhon and Oral (2017)	4. Ederer and Manso (2013)
15. Fischer et al. (2019)	39. Pihie (2007)	5. Kung and Chao (2019)
16. Ford (1999)	40. Susha et al. (2015)	**Meta-analysis**
17. Füller et al. (2012)	41. Thapa et al. (2015)	1. Brandstätter (2011)
18. Galia (2008)	42. Todt et al. (2018)	2. Costa et al. (2015)
19. Gopal and College (2011)	43. van Acker et al. (2018)	3. Kraft and Bausch (2018)
20. Gorozidis and Papaioannou (2016)	44. Vansteenkiste et al. (2005)	4. Ng and Feldman (2013)
21. Hasan et al. (2019)	45. Wang et al. (2018)	5. Park et al. (2004)
22. Hopkins (2016)	46. Weisenfeld and Hauerwaas (2018)	6. Shane et al. (2003)
23. Hosseini and Narayanan (2014)	47. Wu et al. (2013)	
24. Hsu (2009)		
**Interviews**		**Sequential explanatory**
1. Armstrong et al. (2018)	5. Naidoo and Sutherland (2016)	1. Mc Fadden and Gorman (2016)
2. Griffin et al. (2009)	6. Reznickova and Zepeda (2016)	
3. Kandiko (2013)		
4. Minarcine and Shaw (2016)		

## Expectancies in the literature: can I do this?

Innovation stands as an interesting case for EVC as the factors that might potentially motivate innovation are numerous. In terms of the expectancies (self-efficacy and efficacy expectancies; see Bandura, 2001; Wigfield, 1994), persons who see themselves as potentially able to innovate because of an acquired efficacy or confidence should hold a higher expectation of being able to innovate and thus become more invested in the task of innovating. Indeed, EVC and expectancies as a construct have been much clarified by Bandura’s works in explaining the role of self-efficacy (Bandura, 1986, 2001, 2006). Higher investment in the task results in a higher degree of motivation sourced from the self-held conviction that the individual can innovate. An expectancy of success begets well-being, which begets further expectancy of success in innovation and elsewhere. Leaders and managers in organizations assigning simpler innovation tasks early in a program and then building to harder problems according to this logic line should steadily build esteem and self-efficacy.

### Environmental characteristics that build expectancies

Expectancies within the innovation literature were portrayed as being dynamic across contexts and related to individual demographics and personality at least as much as to the environment or situation where innovation was to occur (Monge et al., 1992; Ozorhon & Oral, 2017; Park et al., 2004). The dynamism and relatively differential impact suggest that the current understanding is that there is not one universal strategy for building the confidence to innovate, but rather that the environments need to be informed by the motivational dynamics of the individual and group at hand.

#### Relevant experience and past success

Studies found that environments that facilitated the use of relevant experiences and past successes tended to support innovative behavior due to increased confidence. The effects of experience were varied. It was found in many studies that experience and past success tended to increase the efficacy of other expectancies as collaborations with a mixture of experiences tended to be more fruitful than homogenously experienced teams (Bastian et al., 2018; Bolderdijk et al., 2018; Joy, 2004; Kraft & Bausch, 2018; Kung & Chao, 2019; Mc Fadden & Gorman, 2016; Park et al., 2004; Thapa et al., 2015; Weisenfeld & Hauerwaas, 2018). Similarly, Füller et al. (2012), Chis et al. (2018), and Apergis and Pekka-Economou (2010) found that experience or training in creative settings tended to increase confidence when participating in the innovative process. Another idea in innovation literature is the association of ideas, known as knowledge transfer in other literature, increased confidence and capacity for innovating (Jiang & Thagard, 2014).

Minarcine and Shaw (2016) and Park et al. (2004) found that it was exceedingly rare for people to be adventurous in careers where they had little or no experience. The increase in confidence was dependent on the relatedness between the endeavor the aspirant was pursuing and their relevant experience—the individual’s previous career and the endeavor they wished to undertake. For example, a cutting-edge hairstylist would be more adventurous when going out on their own to open a hair salon; then, they would be starting their own winery. Thus, innovators who had experience related to the endeavor they were considering were much more likely to attempt them. These experiences were found to impart or develop the confidence to be innovative (Armstrong et al., 2018; Chis et al., 2018; Griffin et al., 2009). There was universal agreement that confidence is built through past successes and the validation of past behaviors being met with success (Chi et al., 2018; Griffin et al., 2009; Liu & Chan, 2017; Minarcine & Shaw, 2016; Montani et al., 2014; Park et al., 2004). The research suggests that aspiring innovators are most likely to attempt to innovate when they have relevant experience or are working with peers with relevant experiences.

#### Need supportiveness and stability

Need supportiveness, as coined by Ryan and Deci (2000, 2002, 2017), aims to meet the fullfilment of self-determination and the innate psychological needs of individuals, namely autonomy, competence, and relatedness, as a means of providing a fulfilling environment where they are autonomously motivated as opposed to feeling controlled by external motivation which disenfranchises and often disengages individuals. Need supportiveness of approach and environment was a consistent consideration of literature on promoting innovation and was found to be impactful on the confidence with which individuals undertook innovative endeavors in many disciplines of study, including education (e.g., Kandiko, 2013; Lam et al., 2010), economics and business (e.g., Amabile, 1997; Chaiechi, 2014; Fischer et al., 2019), and creativity (e.g., Wang & Huang, 2015). It was found that organizations that hold holistic views of success that consider employee well-being, personal attainment, autonomy, and company pride, rather than specific outcome measures such as patents, production quotas, or profit, tended to feature more innovative behavior (Kandiko, 2013; Lam et al., 2010). Thus, the environment’s design prioritized fulfilling the innate psychological needs of workers, thus increasing their psychological safety. The availability of support and the feeling that your colleagues would support you if you pursued an innovative endeavor were found to be predictive of self-perceived capacity to innovate (Amabile, 1997; Costa et al., 2015; Delmas & Pekovic, 2018; Ford, 1999; Lettl, 2007; Mc Fadden & Gorman, 2016; Weisenfeld & Hauerwaas, 2018). Stability and consistency, rather than tumult, were found to better support innovative behavior by providing factors that supported the meeting of workers’ innate psychological needs, lending credence to the notion that safety and support are necessary criteria for environments that promote innovation (Chaiechi, 2014; Messmann & Mulder, 2014).

Environments that made aspirants feel that they were safe to make mistakes without severe consequences were those that fulfilled the innate psychological needs as posited by self-determination theory and were found to be especially conducive to innovation confidence (Aarikka-Stenroos et al., 2017; Bastian et al., 2018; Maria Stock et al., 2017; Messmann & Mulder, 2014; Pihie, 2007; Reznickova & Zepeda, 2016). The environments that created the sense of safety were characterized as ones that provided opportunities for workers to pursue passion projects, created stability, gave individuals resources to see their ideas through to completion, and were flexible with methods used to meet goals.

## Values in the literature: a lacking consideration

The thematic analysis of the literature of environments stoking innovation did not reveal many significant value-building capacities, as found in the literature so far in terms of the environment. This is unsurprising as values as a construct in EVC are typically personally held and developed through social contact and intervention in similarly complex tasks; thus, value-building in innovation is much more likely to be found in a systematic review of approaches than one of environments and context. There are a few contributions outside the scope of the review that heighten the salience of value-related factors such as approaches to creating an innovation ecosystem (Audretsch et al., 2019; Carayannis et al., 2018; Smith, 2006) and how to focus on innovator identity development (Arafeh, 2015; Jones et al., 2018) rather than explicit designing of environment.

## Cost in the literature: what is between me and what I want?

Innovation, like other complex tasks, while potentially rewarding, also has contextual material, and psychological costs, such as additional effort, investment of time, pressure, the implications of failure, and loss of both relative stability from the status quo and availability of other options (Flake et al., 2015). For instance, the process of innovating may very well require the investment of additional time and resources to design and operationalize. However, doing things as they have been done in the past does not. The cost of the additional resources may serve to lessen the motivation or diminish the value of innovating. Innovation may place the individual or collaboration under pressure that may be undesirable for some individuals (Flake et al., 2015; Vansteenkiste et al., 2005). Innovation has been portrayed as a risky pursuit because of the possibility of failure, the stigma of being different, and a threat to the status quo (Green, 2013; Lehmann-Ortega & Schoettl, 2005). Even if someone innovates, the idea might not hold the same value to other people. Innovation does have a cost. To some, it constitutes the loss of non-innovative alternatives. To promote innovation development, the expectancies and values must exceed the costs, and the design of environments may facilitate this balancing act.

### Costs in the environment

The study of innovation costs through the environment was scant. Findings in the literature include the dangers of too much competition, the virtues of adopting a cost mitigation strategy, and the effects of fear, pressure, and stress on those aspiring to innovate.

#### Too much competition

Competition in the literature was found to be a cost of innovating (Bessonova & Gonchar, 2017; Bolderdijk et al., 2018; Hasan et al., 2019; Naidoo & Sutherland, 2016). Environments with too high levels of internal competition risk unethical behavior of individuals or groups to succeed, limited knowledge sharing, duplication of efforts, and duplication of spent resources (Kraft & Bausch, 2018; Naidoo & Sutherland, 2016). The external competition also has a drawback on promoting innovation as firms often spent valuable resources differentiating themselves from their competition (de Jong & Flowers, 2018; Hasan et al., 2019; Naidoo & Sutherland, 2016). The results of other studies illustrated that some companies view innovation primarily as a means to escape from the competition, making it a guttural reaction rather than an aspiration (e.g., Bessonova & Gonchar, 2017). These findings point to the need for leaders to moderate the perception of competition, especially internal competition, as moderated levels were found to be helpful tools for stoking the motivation to innovate.

#### Cost mitigation

Costs were sometimes portrayed in the inverse as cost mitigation strategies. These included the additional articulation of the indirect benefits that were found elsewhere in the literature as key motivators. For example, effective strategies to innovation cost mitigation were found to include reasonably priced child care services (Apergis & Pekka-Economou, 2010), safety and harmony in the workplace (Apergis & Pekka-Economou, 2010; Brandstätter, 2011; Chaiechi, 2014; Messmann & Mulder, 2014; Todt et al., 2018), distributing costs as in socialized benefits (Baranchuk et al., 2014; Dietrich et al., 2016; Hopkins, 2016; Hosseini & Narayanan, 2014), and a manager who moderates obstacles (Amabile, 1997; Chen et al., 2019). As an approach for leaders, actively seeking and mitigating the costs of innovating that they identify in their environment is a proactive measure endorsed by findings from the literature.

#### Fear, pressure, and stress

Fear was another common cost faced when attempting an innovative endeavor. Fears offered by the literature included fear of making a product that no one would buy (de Jong & Flowers, 2018; Thapa et al., 2015), risk aversion (Ederer & Manso, 2013; Todt et al., 2018), consequences for failure (Chen et al., 2019; Minarcine & Shaw, 2016), and otherwise existing structures that punish failed or not fully successful attempts at innovation (Minarcine & Shaw, 2016). Only one study gave this fear a face, the status quo (Öberg & Shih, 2014). Innovation runs counter to the inertia of the status quo, making innovation often the more difficult option than maintaining what might be currently done (Ng & Feldman, 2013; Ozorhon & Oral, 2017; Susha et al., 2015). Confronting costs make individuals behave differently (Hsu, 2009; Li & Yu, 2018); leaders seeking to motivate innovative behavior would do well to consider them.

Innovation is portrayed as a stressful endeavor with many different kinds of pressure having an effect including emotional (Jiang & Thagard, 2014), controlling (Ford, 1999; Hsu, 2009), financial (Amabile, 1997; Minarcine & Shaw, 2016), and resource pressures (Aalbers et al., 2013; Amabile, 1997). Pressure was found to originate primarily within organizations as opposed to outside organizations. This finding places the mitigation of pressure squarely within the sphere of influence of leaders to provide adequate resources and a supportive environment to mitigate this factor’s impact.

## Discussion

The synthesized findings illustrate a striking lack of specific and actionable takeaway messages about environments as the literature tends to focus on measurable outcomes rather than the latent considerations that underpin the decisions that aspiring innovators make and the supports and barriers that they consider (see Table 6 for a summary of themes). The literature offers ideas about what motivates innovation, but there has been very little open-ended investigation directly asking innovators what factors motivated them to reach their future goals. This alludes to a need to further investigate individual innovators’ motives as a precursor to a wider investigation of the primary drivers of the aspiration to innovate (e.g., Soleas, 2020). Whereas the absence of consideration of values is almost a given when choosing to focus on the environment rather than approaches and interventions, the paucity of studying costs of innovation in the literature is symptomatic of the primarily positive approach taken by studies, rather than a framework like EVC which also looks at detractive factors like costs. As opposed to many innovation promotion efforts, these findings suggest that efforts to support innovation in various settings, including education and professional development, need to have a deeper understanding of the costs and prices paid by innovators so that they can be mitigated and addressed. It is not enough to provide promotive factors if the underlying costs of innovating remain unaddressed. Additional research is needed to develop and validate approaches and environmental designs that intrinsically and intentionally address innovating costs, perhaps through experimental and intervention designs of studies.

**Table 6:** Summary of research questions and consolidated themes

Research question	Consolidated findings
What is found in the literature about environments that build expectations of success and self-efficacy of individual innovative behavior?	Relevant experience and past success (22 sources) • Experience, past successes, and validation of past behaviors increase confidence in innovating • Mixtures of experience tended to be especially helpful • Training and practice in creative settings was helpful Need supportiveness and stability (24 sources) • Need supportiveness improves confidence • Holistic views of success that considers employee well-being • Support from peers • Stability and safe place to make mistakes
What is found in the literature about environments that build subjective task values for individual innovative behavior?	• No contributions within the scope
What is found in the literature about environments that mitigate the perceived costs of individual innovative behavior?	Too much competition (11 sources) • Internal competition inhibits innovation through limiting knowledge sharing and duplication of efforts and spent resources • External competition can inhibit proactive behaviors Cost mitigation (12 sources) • Psychologically safe environment promotes innovation • Safety net and health benefits make taking risks easier Fear, pressure, and stress (21 sources) • Fear of failure inhibits innovation potential • Avoiding risk and emotional, controlling, financial, and resource pressures makes innovating more difficult

In alignment with the literature, designers of environments should do their utmost to form teams with a mixture of experiences as mixed teams tended to be more productive in their innovating and were found to build individual learners’ confidence. There are promising research grounds for further investigation of the effects of intentionally optimizing the working environment and teams’ organization for these potentially beneficial mixed teams with varied experiences. Similarly, leaders like teachers or supervisors should welcome the utilization of past experiences, particularly those that are likely to be partially transferable to the context at hand. This provides an effective way to build confidence among learners. The literature points to the stability and consistency of environments, as provided by feeling safe and judging success holistically instead of solely by outcomes as being a crucial promoter of innovation. Safety also emerged as an important promoter of innovation in environments where learners felt safe to make mistakes. Leaders in a position to influence the environment can encourage the pursuit of passion projects and develop novel approaches by ensuring that it is reasonably acceptable to make mistakes. In short, an environment that encourages the use of past experiences and feels stable and safe was found by the literature to promote innovation. Future research should be conducted to further illustrate the designs of environments and approaches that facilitate psychological safety.

It was unsurprising that value building was conspicuously lacking from the literature concerning environments as value building tends to be a personal and socially driven process rather than an environment-driven process without intervention (Barron & Hulleman, 2015). Further research should be conducted to verify that this is not an artifact of the search strategy, and the approaches that heighten the value of entrepreneurship are not in actuality based on the design of environments.

Literature tended to portray the costs of innovation as something that innovators avoided. However the identified costs hint at the hindering factors that could make innovation more likely if they were to be addressed and mitigated. Environments that were less internally competitive were found to better promote innovation than those with higher levels of internal competition. External competition was found to be promotive. Influencers of the environment could make innovation more likely by ensuring that learning and working environments would focus on competition external rather than internal to the institution.

Other key considerations to mitigate costs included providing indirect benefits like health insurance and subsidizing childcare as this proactively eliminated costs and time-drains that would distract workers and learners from their work and potential innovating. Leaders seeking to make innovation more likely should proactively mitigate these costs. This same principle applies to the perceived fears, pressures, and stresses that occur within workplaces and learning spaces, which leaders could counter by having a reasonable tolerance for failure and focusing initially on educational rather than punitive responses to non-optimal outcomes.

### Delimitations and limitations

The ambiguous and wide-ranging language of innovation made searching for articles inherently tricky as articles on related topics could evade identification by using words that are not strictly synonymous in definition (e.g., entrepreneurship) but certainly adjacent in interest. In interpreting the findings, it is clear that environmental considerations are not the only factors that impact innovating. An approach considering only environmental factors underestimates the literature findings that innate and personality traits and the strategies and approaches of teachers and other supervisory figures can have on promoting innovation (Soleas, 2020). Rather, innate factors and interpersonal interactions are greatly important and should be considered in tandem with environmental considerations.

## Conclusion

As also shown in a systematic review of strategies, it is clear that there is an unbalanced primacy in the innovation literature favoring the study of business, entrepreneurship, and corporate environments with emergent representation from environments in the arts, educational, and social justice sectors. This creates a situation where conversations and conceptualizations about motivating innovation are dominated by business and corporate considerations instead of more representatively including strong contributions from humanities, sciences, and social sciences. There is ample opportunity for integration with entrepreneurship studies leading to scholarly synergy between these two related fields. Additional study is needed that works with interdisciplinary samples that also gather deep narrative and case-driven data that considers the experiences of innovators and organizations. Although there are clusters of journals, namely in business contexts, with emerging hubs in psychology, the pool of authors contributing to the knowledge of innovation is diffuse, meaning that more disciplines contribute to our knowledge of the environments that stoke innovation.

There is also a common trend of using surveys with homogenous groups of individuals within a single discipline, while interviews are rare. In terms of methodology, the field is dominated by surveys of employees in business settings rather than of bonafide innovators. This focus on survey research precludes the possibility of having concrete details and rich articulation of narration from innovators on their thinking. Survey methodology, while a useful tool of scholarship, does not provide deep narrative and personal understanding in the same way that interview, focus group, or other open-ended question-driven methodologies could. As well, the relative lack of interdisciplinary participants should be concerning as it means that studies are still largely disinterested in cross-disciplinary efforts to make innovation more likely, hinting at a continuance of siloed efforts to promote innovation. Although there is an emergent field of study on innovation education, this field has yet to consider the unique motivation of aspiring innovators as foretold by existing innovators’ experiences.

## Data Availability

Data sharing does not apply to this article as no datasets were generated or analyzed during the current study. However, lists of analyzed articles are available in the manuscript in the tables and the references.

## Abbreviation

EVC: Expectancy-Value-Cost Theory (Barron and Hulleman 2015)—A major theory of motivation that theorizes that the motivation for a task is the function of the expectancies (expectations of success, confidence, self-efficacy) and the values (a direct or indirect, tangible, or intangible benefit for performing the task) balanced against the perceived costs of performing the task.

## References

[CR1] Aalbers R, Dolfsma W, Koppius O (2013). Individual connectedness in innovation networks: On the role of individual motivation. Research Policy.

[CR2] Aarikka-Stenroos L, Jaakkola E, Harrison D, Mäkitalo-Keinonen T (2017). How to manage innovation processes in extensive networks: A longitudinal study. Industrial Marketing Management.

[CR3] Amabile TM (1997). Motivating creativity in organizations: on doing what you love and loving what you do. California Management Review.

[CR4] Apergis N, Pekka-Economou V (2010). Incentives and female entrepreneurial activity: Evidence from panel firm level data. International Advances in Economic Research.

[CR5] Arafeh, L. (2015). An entrepreneurial key competencies’ model. *Journal of Innovation and Entrepreneurship*, 5(1). 10.1186/s13731-016-0048-6.

[CR6] Armstrong A, van der Lingen E, Lourens R, Chen JY-J (2018). Towards a new model of grit within a cognitive-affective framework of self-regulation. South African Journal of Business Management.

[CR7] Audretsch DB, Cunningham JA, Kuratko DF, Lehmann EE, Menter M (2019). Entrepreneurial ecosystems: Economic, technological, and societal impacts. Journal of Technology Transfer.

[CR8] Bandura, A. (1986). The explanatory and predictive scope of self-efficacy theory. *Journal of Social and Clinical Psychology*, *4*(3), 359–373.

[CR9] Bandura A (2001). Social cognitive theory: An agentic perspective. Annual Review of Psychology.

[CR10] Bandura A (2006). Toward a psychology of human agency. Perspectives on Psychological Science.

[CR11] Baranchuk N, Kieschnick R, Moussawi R (2014). Motivating innovation in newly public firms. Journal of Financial Economics.

[CR12] Baregheh A, Rowley J, Sambrook S (2009). Towards a multidisciplinary definition of innovation. Management Decision.

[CR13] Barron, K. E., & Hulleman, C. S. (2015). Expectancy-Value-Cost model of motivation. In J. S. Eccles & K. Salmelo-Aro (Eds.), *International encyclopedia of social and behavioral sciences* (2nd ed.). London: Elsevier.

[CR14] Bastian B, Jetten J, Thai HA, Steffens NK (2018). Shared adversity increases team creativity through fostering supportive interaction. Frontiers in Psychology.

[CR15] Bergendahl, M., Magnusson, M., & Björk, J. (2015). Ideation high performers: a study of motivational factors. *Creativity Research Journal, 27*(4), 361–368. 10.1080/10400419.2015.1088266.

[CR16] Bessonova E, Gonchar K (2017). Incentives to innovate in response to competition: The role of agency costs. Economic Systems.

[CR17] Bolderdijk JW, Brouwer C, Cornelissen G (2018). When do morally motivated innovators elicit inspiration instead of irritation?. Frontiers in Psychology.

[CR18] Brandstätter H (2011). Personality aspects of entrepreneurship: A look at five meta-analyses. Personality & Individual Differences.

[CR19] Brcic, J. (2010). Motivational profile of astronauts at the International Space Station. *Acta Astronautica, 67*(9–10), 1110–1115. 10.1016/j.actaastro.2010.06.044.

[CR20] Carayannis EG, Grigoroudis E, Campbell DFJ, Meissner D, Stamati D (2018). The ecosystem as helix: an exploratory theory-building study of regional co-opetitive entrepreneurial ecosystems as Quadruple/Quintuple Helix Innovation Models. R and D Management.

[CR21] Chaiechi T (2014). The broken window: Fallacy or fact– A Kaleckian–Post Keynesian approach. Economic Modelling.

[CR22] Chen JX, Sharma P, Zhan W, Liu L (2019). Demystifying the impact of CEO transformational leadership on firm performance: Interactive roles of exploratory innovation and environmental uncertainty. Journal of Business Research.

[CR23] Chi M, Wang W, Lu X, George JF (2018). Antecedents and outcomes of collaborative innovation capabilities on the platform collaboration environment. International Journal of Information Management.

[CR24] Chis AE, Moldovan A-N, Murphy L, Pathak P, Hava Muntean C (2018). Investigating flipped classroom and problem-based learning in a programming module for computing conversion course. Journal of Educational Technology & Society.

[CR25] Cordero, R., Walsh, S. T., & Kirchhoff, B. A. (2005). Motivating performance in innovative manufacturing plants. *Journal of High Technology Management Research, 16*(1), 89–99. 10.1016/j.hitech.2005.06.005.

[CR26] Costa S, Páez D, Sánchez F, Garaigordobil M, Gondim S (2015). Personal factors of creativity: A second order meta-analysis. Journal of Work and Organizational Psychology.

[CR27] Curran, B., & Walsworth, S. (2014). Can you pay employees to innovate? Evidence from the Canadian private sector. *Human Resource Management Journal, 24*(3), 290–306. 10.1111/1748-8583.12036.

[CR28] de Jong JPJJ, Flowers S (2018). Free in, free out? Outbound transfer of user innovations in small UK firms. Industrial Marketing Management.

[CR29] Delmas MA, Pekovic S (2018). Corporate sustainable innovation and employee behavior. Journal of Business Ethics.

[CR30] Demircioglu, M. A., & Audretsch, D. B. (2017). Conditions for innovation in public sector organizations. *Research Policy, 46*(9), 1681–1691. 10.1016/j.respol.2017.08.004.

[CR31] Dietrich M, Znotka M, Guthor H, Hilfinger F (2016). Instrumental and non-instrumental factors of social innovation adoption. Voluntas.

[CR32] Duverger, P. (2012). Variety is the spice of innovation: mediating factors in the service idea generation process. *Creativity and Innovation Management, 21*(1), 106–119. 10.1111/j.1467-8691.2011.00621.x.

[CR33] Ederer F, Manso G (2013). Is pay for performance detrimental to innovation?. Management Science.

[CR34] Fernandez, S., & Pitts, D. W. (2011). Understanding employee motivation to innovate: evidence from front line employees in United States federal agencies. *Australian Journal of Public Administration, 70*(2), 202–222. 10.1111/j.1467-8500.2011.00726.x.

[CR35] Fischer C, Malycha CP, Schafmann E (2019). The influence of intrinsic motivation and synergistic extrinsic motivators on creativity and innovation. Frontiers in Psychology.

[CR36] Flake JK, Barron KE, Hulleman C, McCoach DB, Welsh ME, McCoach BD, Welsh ME (2015). Measuring cost: The forgotten component of expectancy-value theory. Contemporary Educational Psychology.

[CR37] Ford CM (1999). Interpretive style, motivation, ability and context as predictors of executives’ creative performance. Creativity and Innovation Management.

[CR38] Füller J, Matzler K, Hutter K, Hautz J (2012). Consumers’ creative talent: Which characteristics qualify consumers for open innovation projects? An exploration of asymmetrical effects. Creativity and Innovation Management.

[CR39] Galia, F. (2008). Intrinsic-extrinsic motivations and knowledge sharing in French firms. *ICFAI Journal of Knowledge Management, 6*(1), 56–80.

[CR40] Gopal, G., & College, E. (2011). Using reward systems to motivate employees for innovation. Niall Hoarty, Medtronic Corporation, Galway, Ireland Dr. Gurram Gopal, Elmhurst College, Elmhurst, IL Dr. Laurence P. Elwood, Galway-Mayo Institute of Technology, Galway, Ireland. *Global Education Journal*, 56–67.

[CR41] Gorozidis, G. S., & Papaioannou, A. G. (2016). Teachers’ achievement goals and self-determination to engage in work tasks promoting educational innovations. *Learning and Individual Differences, 49*, 46–58. 10.1016/j.lindif.2016.05.014.

[CR42] Green, E. (2013, June 20). *Innovation: The history of a buzzword*, (p. 20). The Atlantic. http://www.theatlantic.com/business/archive/2013/06/innovation-the-history-of-a-buzzword/277067/

[CR43] Griffin A, Price RL, Maloney MM, Vojak BA, Sim EW (2009). Voices from the field: How exceptional electronic industrial innovators innovate. Journal of Product Innovation Management.

[CR44] Hartmann, A. (2006). The role of organizational culture in motivating innovative behaviour in construction firms. *Construction Innovation, 6*(3), 159–172. 10.1108/14714170610710712.

[CR45] Hasan R, Lowe B, Petrovici D (2019). An empirical comparison of consumer innovation adoption models: Implications for subsistence marketplaces. Journal of Public Policy & Marketing.

[CR46] Heyvaert M, Hannes K, Maes B, Onghena P (2013). Critical appraisal of mixed methods studies. Journal of Mixed Methods Research.

[CR47] Hopkins V (2016). Institutions, incentives, and policy entrepreneurship. Policy Studies Journal.

[CR48] Hosseini SMP, Narayanan S (2014). Adoption, adaptive innovation, and creative innovation among SMEs in Malaysian manufacturing. Asian Economic Papers.

[CR49] Hsu Y (2009). Exploring design innovation and performance: The roles of issue related to design strategy. Journal of Engineering Design.

[CR50] Jermias, J. (2007). The effects of corporate governance on the relationship between innovative efforts and performance. *European Accounting Review, 16*(4), 827–854. 10.1080/09638180701707045.

[CR51] Jiang M, Thagard P (2014). Creative cognition in social innovation. Creativity Research Journal.

[CR52] Johannessen J-A, Olsen B, Lumpkin GT (2001). Innovation as newness: What is new, how new, and new to whom?. European Journal of Innovation Management.

[CR53] Jones P, Klapper R, Ratten V, Fayolle A (2018). Emerging themes in entrepreneurial behaviours, identities and contexts. International Journal of Entrepreneurship and Innovation.

[CR54] Joy S (2004). Innovation motivation: The need to be different. Creativity Research Journal.

[CR55] Kandiko CB (2013). Leadership and creativity in higher education: The role of interdisciplinarity (H05). London Review of Education.

[CR56] Koloniari, M., Vraimaki, E., & Fassoulis, K. (2018). Fostering innovation in academic libraries through knowledge creation. *Journal of Academic Librarianship, 44*(6), 793–804. 10.1016/j.acalib.2018.09.016.

[CR57] Kraft PS, Bausch A (2018). Managerial social networks and innovation: A meta-analysis of bonding and bridging effects across institutional environments. Journal of Product Innovation Management.

[CR58] Kung FYH, Chao MM (2019). The impact of mixed emotions on creativity in negotiation: An interpersonal perspective. Frontiers in Psychology.

[CR59] Lam S, Cheng RW, Choy HC (2010). School support and teacher motivation to implement project-based learning. Learning & Instruction.

[CR60] Lehmann-Ortega L, Schoettl J-M (2005). From buzzword to managerial tool: The role of business model in strategic innovation. Paper presented at the CLADEA annual assembly.

[CR61] Lerner, J., & Wulf, J. (2018). Innovation and incentives: evidence from corporate R&D. *The Review of Economics and Statistics, 89*(4), 634–644.

[CR62] Lettl C (2007). User involvement competence for radical innovation. Journal of Engineering & Technology Management.

[CR63] Li J, Yu D (2018). The path to innovation: The antecedent perspective of intellectual capital and organizational character. Frontiers in Psychology.

[CR64] Liberati A, Altman DG, Tetzlaff J, Mulrow C, Gøtzsche PC, Ioannidis JPA, Clarke M, Devereaux PJ, Kleijnen J, Moher D (2009). The PRISMA statement for reporting systematic reviews and meta-analyses of studies that evaluate health care interventions: explanation and elaboration. PLoS Medicine.

[CR65] Liu A, Chan I (2017). Critical role of the learning transfer climate in fostering innovation in construction. Journal of Management in Engineering.

[CR66] Lukoschek, C. S., Gerlach, G., Stock, R. M., & Xin, K. (2018). Leading to sustainable organizational unit performance: Antecedents and outcomes of executives’ dual innovation leadership. *Journal of Business Research, 91*(December 2017), 266–276. 10.1016/j.jbusres.2018.07.003.

[CR67] Mack, T., & Landau, C. (2015). Winners, losers, and deniers: Self-selection in crowd innovation contests and the roles of motivation, creativity, and skills. *Journal of Engineering and Technology Management, 37*, 52–64. 10.1016/j.jengtecman.2015.08.003.

[CR68] Manimala, M. J., Jose, P. D., & Thomas, K. R. (2006). Organizational constraints on innovation and intrapreneurship: insights from public sector. *Vikalpa: The Journal for Decision Makers, 31*(1), 49–60. 10.1177/0256090920060104.

[CR69] Maria Stock R, Zacharias NA, Schnellbaecher A (2017). How do strategy and leadership styles jointly affect co-development and its innovation outcomes?. Journal of Product Innovation Management.

[CR70] Mc Fadden T, Gorman M (2016). Exploring the concept of farm household innovation capacity in relation to farm diversification in policy context. Journal of Rural Studies.

[CR71] Messmann G, Mulder RH (2014). Exploring the role of target specificity in the facilitation of vocational teachers’ innovative work behaviour. Journal of Occupational and Organizational Psychology.

[CR72] Minarcine S, Shaw C (2016). Motivations for entrepreneurship. International Journal of the Academic Business World.

[CR73] Monge PR, Cozzens MD, Contractor NS (1992). Communication and motivational predictors of the dynamic of organizational innovation. Organization Science.

[CR74] Montani F, Odoardi C, Battistelli A (2014). Individual and contextual determinants of innovative work behaviour: Proactive goal generation matters. Journal of Occupational and Organizational Psychology.

[CR75] Nager A, Hart D, Ezell S, Atkinson RD (2016). The demographics of innovation in the United States.

[CR76] Naidoo S, Sutherland M (2016). A management dilemma: Positioning employees for internal competition versus internal collaboration. Is coopetition possible?. South African Journal of Business Management.

[CR77] Ng TWH, Feldman DC (2013). Does longer job tenure help or hinder job performance?. Journal of Vocational Behavior.

[CR78] Öberg C, Shih TTY (2014). Divergent and convergent logic of firms: Barriers and enablers for development and commercialization of innovations. Industrial Marketing Management.

[CR79] Ozorhon B, Oral K (2017). Drivers of innovation in construction projects. Journal of Construction Engineering and Management.

[CR80] Park M, Nepal MP, Dulaimi MF (2004). Dynamic modeling for construction innovation. Journal of Management in Engineering.

[CR81] Pihie ZAL (2007). An analysis of academic experience to develop entrepreneurial attributes and motivation among at-risk students. The International Journal of Learning.

[CR82] Pihlajamaa, M. (2017). Going the extra mile: managing individual motivation in radical innovation development. *Journal of Engineering and Technology Management, 43, *48–66. 10.1016/j.jengtecman.2017.01.003.

[CR83] Reznickova A, Zepeda L (2016). Can self-determination theory explain the self-perpetuation of social innovations? A case study of slow food at the University of Wisconsin-Madison. Journal of Community & Applied Social Psychology.

[CR84] Ryan RM, Deci EL (2000). Self-determination theory and the facilitation of intrinsic motivation, social development, and well-being. American Psychologist.

[CR85] Ryan, R. M., & Deci, E. L. (2002). An overview of self-determination theory: An organismic-dialectical perspective. In E. L. Deci & R. M. Ryan (Eds.), *Handbook of self-determination research* (Vol. 55, Issue 1, pp. 3–35). Rochester: University of Rochester Press.

[CR86] Ryan, R. M., & Deci, E. L. (2017). *Self-determination theory: Basic psychological needs in motivation, development, and wellness*. New York: The Guilford Press.

[CR87] Shane, S., Locke, E. A., & Collins, C. J. (2003). Entrepreneurial motivation. *Human Resource Management Review, 13*(2), 257–279. 10.1016/S1053-4822(03)00017-2.

[CR88] Smith KR (2006). Building an innovation ecosystem: Process, culture and competencies. Industry and Higher Education.

[CR89] Soleas EK (2018). Get off my lawn: Why capitalism’s monopoly on innovation is bad for us all and what educators can do about it. Graduate Student Symposium Selected Papers.

[CR90] Soleas, E. K. (2020). Expectancies, values, and costs of innovating identified by Canadian innovators: A motivational basis for supporting innovation talent development. *Journal of Advanced Academics*, 1–25. 10.1177/1932202X20904772.

[CR91] Susha I, Grönlund A, Janssen M (2015). Driving factors of service innovation using open government data: An exploratory study of entrepreneurs in two countries. Information Polity.

[CR92] Thapa BEP, Niehaves B, Seidel CE, Plattfaut R (2015). Citizen involvement in public sector innovation: Government and citizen perspectives. Information Polity.

[CR93] The Campbell Collaboration (2017). Campbell systematic reviews: Policies and guidelines. Campbell Policies and Guidelines Series No. 1.

[CR94] Todt G, Weiss M, Hoegl M (2018). Mitigating negative side effects of innovation project terminations: The role of resilience and social support. Journal of Product Innovation Management.

[CR95] U.S. Department of Education (2014). What do we mean by “innovation”? About ed.

[CR96] van Acker, W., Wynen, J., & Op de Beeck, S. (2018). Illuminating the Gender Divide in Public Sector Innovation: Evidence From the Australian Public Service. *Public Personnel Management, 47*(2), 175–194. 10.1177/0091026017747299.

[CR97] Vansteenkiste M, Lens W, Witte H, Feather NT (2005). Understanding unemployed people’s job search behaviour, unemployment experience and well-being: A comparison of Expectancy-Value Theory and Self-Determination Theory. British Journal of Social Psychology.

[CR98] Wang G, Huang H (2015). Effect of Chinese employees’ emotional creativity on their innovative performance. Social Behavior and Personality.

[CR99] Wang, Y., Liu, J., Zhu, Y., & Bastian, B. (2018). Humble leadership, psychological safety, knowledge sharing, and follower creativity: a cross-level investigation. *Frontiers in Psychology, 9*(Article 1727), 1–9. 10.3389/fpsyg.2018.0172. 10.3389/fpsyg.2018.01727 PMC6156152 30283379

[CR100] Weisenfeld U, Hauerwaas A (2018). Adopters build bridges: Changing the institutional logic for more sustainable cities. From action to workset to practice. Research Policy.

[CR101] Wigfield A (1994). Expectancy-Value Theory of achievement motivation: A developmental perspective. Educational Psychology Review.

[CR102] Wu, A., Su, J., & Wang, H. (2013). Internal innovation or external innovation? An organizational context-based analysis in China. *Journal of High Technology Management Research, 24*(2), 118–129. 10.1016/j.hitech.2013.09.006.

